# Rab20 Regulates Phagosome Maturation in RAW264 Macrophages during Fc Gamma Receptor-Mediated Phagocytosis

**DOI:** 10.1371/journal.pone.0035663

**Published:** 2012-04-24

**Authors:** Youhei Egami, Nobukazu Araki

**Affiliations:** Department of Histology and Cell Biology, School of Medicine, Kagawa University, Miki, Kagawa, Japan; University of Geveva, Switzerland

## Abstract

Rab20, a member of the Rab GTPase family, is known to be involved in membrane trafficking, however its implication in FcγR-mediated phagocytosis is unclear. We examined the spatiotemporal localization of Rab20 during phagocytosis of IgG-opsonized erythrocytes (IgG-Es) in RAW264 macrophages. By the live-cell imaging of fluorescent protein-fused Rab20, it was shown that Rab20 was transiently associated with the phagosomal membranes. During the early stage of phagosome formation, Rab20 was not localized on the membranes of phagocytic cups, but was gradually recruited to the newly formed phagosomes. Although Rab20 was colocalized with Rab5 to some extent, the association of Rab20 with the phagosomes persisted even after the loss of Rab5 from the phagosomal membranes. Then, Rab20 was colocalized with Rab7 and Lamp1, late endosomal/lysosomal markers, on the internalized phagosomes. Moreover, our analysis of Rab20 mutant expression revealed that the maturation of phagosomes was significantly delayed in cells expressing the GDP-bound mutant Rab20-T19N. These data suggest that Rab20 is an important component of phagosome and regulates the phagosome maturation during FcγR-mediated phagocytosis.

## Introduction

Phagocytosis, a specialized form of endocytosis characterized by ingestion of large particles (>1 µm in diameter), plays an important role in innate immunity and in tissue remodeling. Professional phagocytes, mainly macrophages and neutrophils, are equipped for ingesting foreign particles, invading microorganisms and apoptotic cells, contributing to the resolution of infections and the clearance of senescence and damaged cells. Phagocytosis of a particle is initiated by the binding of the particle to a specific cell surface receptor. A large number of receptors, such as Fc γ receptors (FcγRs), complement receptors (CRs) and Toll-like receptors (TLRs), have been implicated in this process [1]. Phagocytosis via FcγRs, which has been the best characterized, is accompanied by actin polymerization and reorganization, which drive the formation of phagocytic cups around IgG-opsonized particles [2]–[5]. After the closure of the phagocytic cups, the intracellular vacuoles termed phagosomes mature via a series of interaction with endocytic compartments and eventually fuse with lysosomes for particle degradation [6], [7].

Rab proteins constitute the largest family of monomeric small GTPases that function as molecular switches by cycling between their inactive GDP- and active GTP-bound forms [8]–[10]. The inactive GDP-bound form is thought to be quiescence in the cytoplasm and is complexed with their chaperone Rab-GDIs. Conversely, GTP-bound forms physically interact with effectors on membranes and regulate membrane trafficking. To date, a number of Rab GTPases have been shown to be involved in vesicle formation, motility, docking and fusion [11]–[14]. Lines of evidence indicate that Rab GTPases are required for the interaction of phagosomes with endocytic and lysosomal vesicles [7]. Rab5 and Rab7 are the best-characterized Rab proteins with regard to their localization to phagosomes and their participation in maturation during FcγR-mediated phagocytosis [15]. Rab5 regulates the fusion of nascent phagosomes with sorting endosomes and is important for the acquisition of Rab7. Rab7 allows phagosomes to interact with late endosomes and lysosomes, leading to the formation of phagolysosomes. Moreover, other Rab GTPases, such as Rab23, Rab35 and Rab10, have been detected on the phagosomal membranes [16]–[18]. During FcγR-mediated phagocytosis, these Rab GTPases have been shown to control phagosome maturation [16], [17]. To date, a growing number of Rab GTPases have been identified on phagosomal membranes [17], [19], [20]. However, most of them have not been studied in terms of FcγR-mediated phagocytosis and phagolysosome biogenesis. One such member is Rab20, which is mainly expressed in the apical side of kidney tubule, intestine epithelial cells and non-polarized cells [21], [22]. It is known that Rab20 localizes in the Golgi, ER region and some endocytic compartments, however its function remains uncharacterized [21], [23], [24]. Most recently, it has been shown that Rab20 is recruited to phagolysosomes containing *Staphylococcus aureus*, whereas it is dissociated from phagosomes containing *Mycobacterium tuberculosis*, which arrests phagosome maturation [25]. These data suggest that Rab20 seems to be involved in phagosome maturation in bacteria-infected macrophages. Thus, it is of our interest to investigate whether or not Rab20 is involved in FcγR-mediated phagocytosis. In the present study, we revealed that Rab20 is transiently associated with FcγR-mediated phagosomes and regulates phagolysosome formation in macrophages. This study provides the first evidence that Rab20 is a novel component of the FcγR-mediated phagosomal maturation machinery.

## Results

### Rab20 is associated with phagosomal membrane during Fcγ-receptor mediated phagocytosis in macrophages

**Figure 1:**
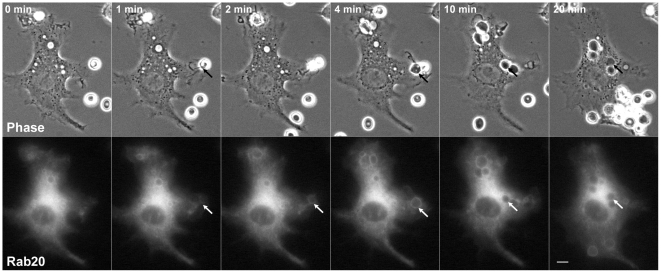
Live-cell imaging of GFP-Rab20 in RAW264 macrophages during phagocytosis of IgG-Es. Live RAW264 cells expressing GFP-Rab20 were put into contact with IgG-Es and observed by phase-contrast and fluorescence microscopy. Time-lapse images were acquired using the MetaMorph imaging system. Phase-contrast images are shown (upper panels). The elapsed time is indicated at the top. The binding of IgG-Es to the cell surface was set as time 0. It is noteworthy that Rab20 was associated with newly formed phagosomes (arrows). At least four cells were examined in four independent experiments. Similar results were obtained from four independent experiments. Scale bars: 5 µm. The corresponding video is Movie S1.

Rab20 has been shown to be expressed in polarized epithelial cells [21] and was also detected in macrophages by RT-PCR analysis (Figure S1). To examine whether or not Rab20 is involved in FcγR-mediated phagocytosis, we observed the localization of Rab20 during the process of IgG erythrocytes (IgG-Es) engulfment in live RAW264 macrophages expressing GFP-Rab20 by phase-contrast and fluorescence microscopy. Prior to the beginning of phagocytosis, Rab20 was diffusely localized in the cytosol and somewhat enriched in the perinuclear region, as previously shown in other cell types [21], [23], [24]. Although Rab20 was scarcely observed on the sites of IgG-E binding or on the membrane of phagocytic cups along the surface of IgG-Es, Rab20 was gradually recruited to newly formed phagosomes after closing phagocytic cups into phagosomes (Figure 1 and Movie S1). The levels of Rab20 in the phagosomal membranes peaked at around 5 min after internalization of IgG-E. Subsequently, Rab20 dissociated from the phagosomes over time. These findings indicate that Rab20 is transiently associated with phagosome during FcγR-mediated phagocytosis in macrophages.

### The association of Rab20 with phagosomal membranes is preceded by that of Rab5 and followed by those of Rab7 and Lamp1

**Figure 2:**
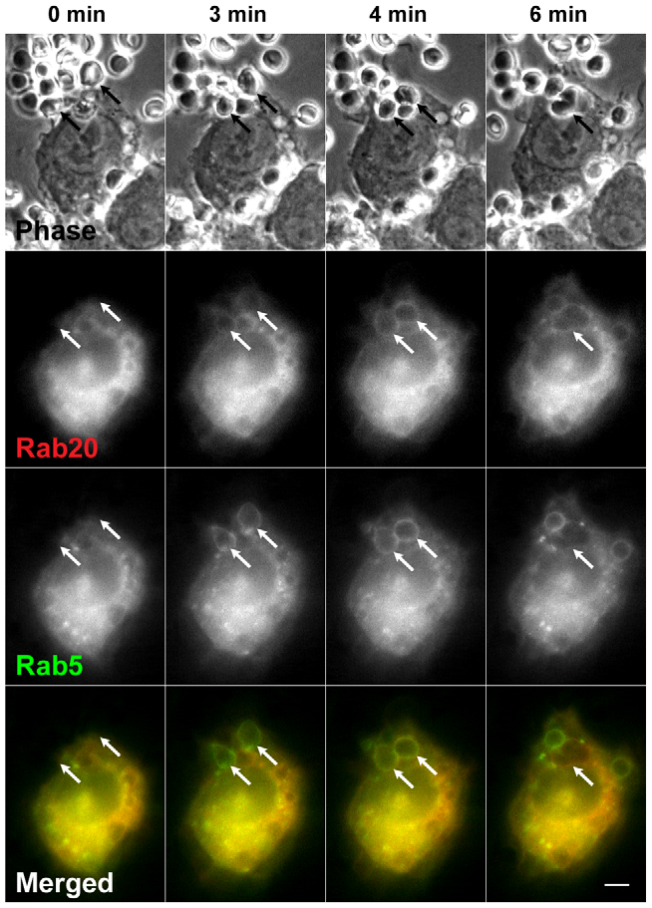
The association of Rab20 with phagosomal membranes persists following the loss of Rab5. Live RAW264 cells co-expressing CFP-Rab20 (red) and GFP- Rab5 (green) were incubated with IgG-Es and monitored by phase-contrast and fluorescence microscopy. The elapsed time is indicated at the top. The binding of IgG-Es to the cell surface was set as time 0. It was found that Rab20 and Rab5 were transiently colocalized on formed phagosomes and that Rab20 remained associated with phagosomal membrane from which Rab5 was dissociated (arrows). Representative images from three independent experiments are shown. Scale bar: 5 µm. The corresponding video is Movie S2.

Rab5 has been known to be temporarily associated with phagosomes in macrophages [3], [15]. To clarify the stage of Rab20 recruitment to nascent phagosomes, we co-transfected RAW264 cells with CFP-Rab20 and GFP-Rab5, and observed their localization during phagocytosis of IgG-Es. Before the onset of phagocytosis, Rab20 and Rab5 were not localized in the plasma membrane (Figure 2). Noteworthy, time-lapse imaging showed that the recruitment of Rab20 to phagosomes occurred later than that of Rab5 during phagosome formation (Figure 2 and Movie S2). Subsequently, Rab20 was transiently colocalized with Rab5 on newly formed phagosomes. In the early maturation stage of phagosomes, Rab5 was dissociated from the phagosomal membranes within 6 min. However, Rab20 remained associated with them over an extended time period.

**Figure 3:**
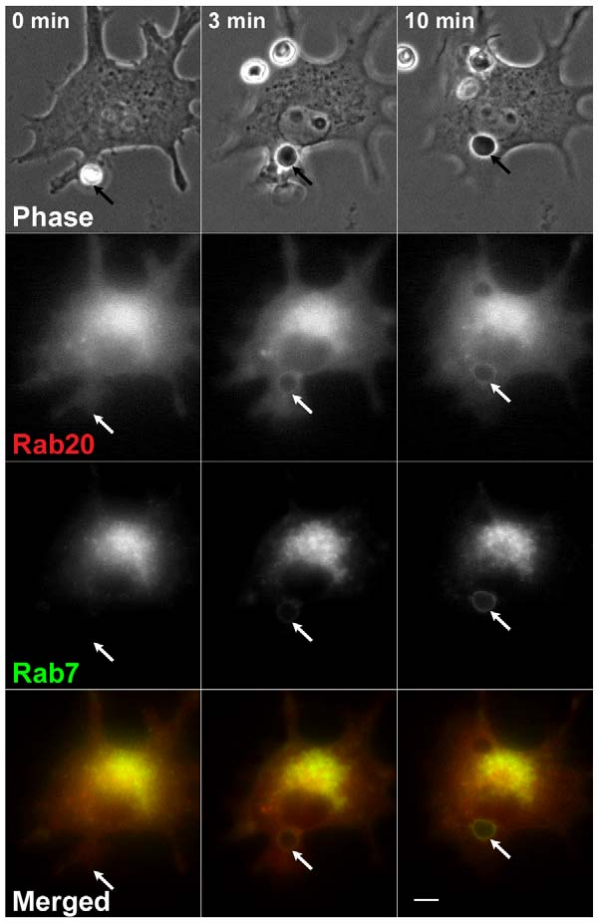
Live-cell imaging of Rab20 and Rab7 in RAW264 macrophages during phagocytosis of IgG-Es. RAW264 macrophages co-expressing CFP-Rab20 (red) and YFP-Rab7 (green) were allowed to interact with IgG-Es and observed by phase-contrast and fluorescence microscopy. Although Rab20 was colocalized with Rab7 on phagosomes, the recruitment of Rab20 to phagosomes occurred earlier than that of Rab7 (arrows). Similar results were obtained from four independent experiments. Scale bar: 5 µm. The corresponding video is Movie S3.

**Figure 4:**
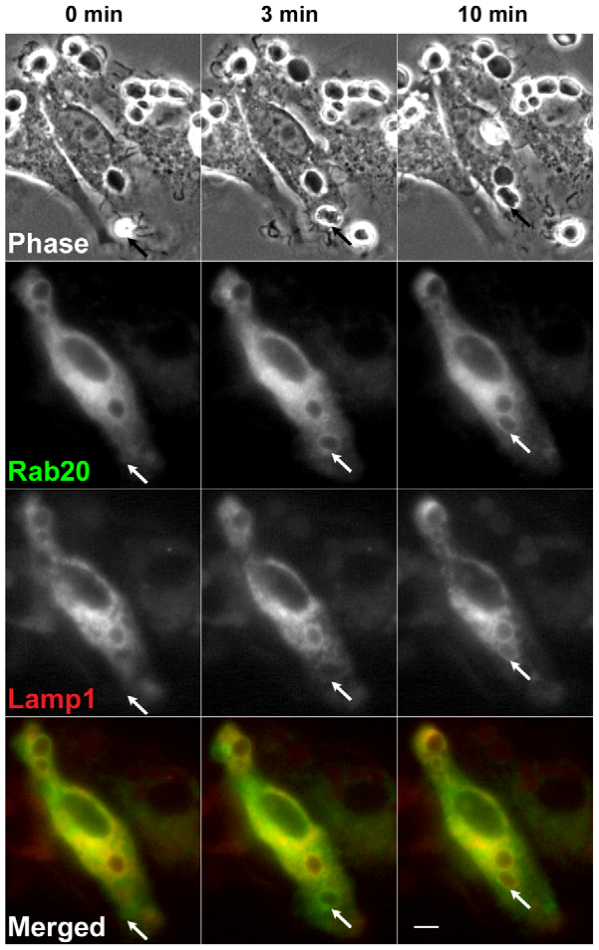
Time-lapse imaging showing the spatiotemporal relationship between Rab20 and Lamp1 during phagocytosis. RAW264 cells were co-transfected with GFP-Rab20 (green) and CFP-Lamp1 (red). Time-lapse images of phase-contrast, GFP-Rab20 and CFP-Lamp1 were taken by phase-contrast and fluorescence microscopy. The association of Rab20 with phagosomes was followed by Lamp1 accumulation (arrows). Similar results were obtained from fourindependent experiments. Scale bar: 5 µm. The corresponding video is Movie S4.

It has been reported that phagosomes sequentially acquire Rab5 (an early endosomal marker), Rab7 and Lamp1 (late endosomal and lysosomal markers) during phagosome maturation [3], [15]. To compare the distribution of Rab20 and Rab7 in the later stage of phagocytosis, RAW264 macrophages were co-transfected with YFP-Rab7 and CFP-Rab20 and were observed by phase-contrast and fluorescence microscopy. Time-lapse images in Figure 3 revealed that, in the initial stage of phagosome maturation, moderate levels of Rab20 were present on the phagosomal membrane where Rab7 was scarcely observed. In the intermediate/late stage of phagosome maturation, Rab20 and Rab7 were largely colocalized on maturing phagosomes (Movie S3). Time-lapse analysis of live cells co-expressing GFP-Rab20 and CFP-Lamp1 also showed that Rab20 is recruited to nascent phagosomes before Lamp1 accumulation in the phagosomal membranes. Then, Rab20 seemed to be associated with Lamp1-positive phagosomes for a while (Figure 4 and Movie S4).

To evaluate the changes in Rab5, Rab20, Rab7 and Lamp1 levels on individual phagosomes, quantitative image analysis of fluorescent protein fusions was performed. As shown in Figure S2, the levels of Rab5, Rab20, Rab7 and Lamp1 peaked at around 2–4, 4–6, 8–12 and 8–12 min, respectively. Taken together, these results indicate that Rab20 is recruited to phagosomal membranes later than Rab5 and earlier than Rab7 and Lamp1.

### The expression of GDP-bound mutant of Rab20 inhibits phagosome maturation during FcγR-mediated phagocytosis

**Figure 5:**
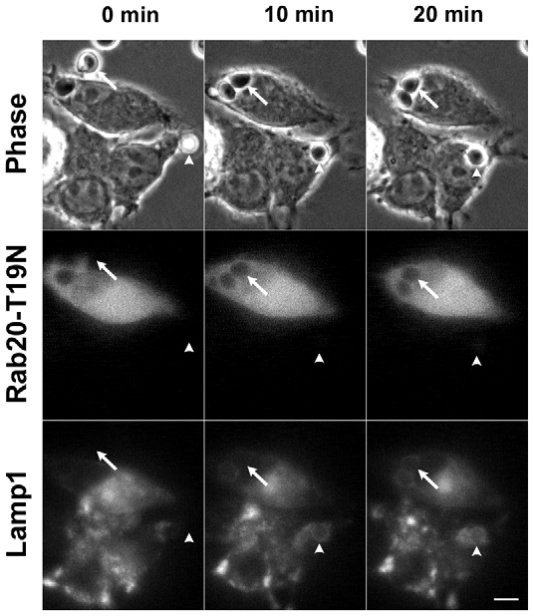
Time-lapse images showing different dynamics of Lamp1 between a Rab20-T19N-expressing cell and a non-expressing cell during ingestion of IgG-Es. RAW264 macrophages co-expressing GFP-Rab20-T19N and CFP-Lamp1 were exposed to IgG-Es and observed by phase-contrast and fluorescence microscopy. The delivery of Lamp1 to formed phagosomes was inhibited in cells expressing Rab20-T19N (arrows) as compared to non-expressing controls (arrowheads). Similar results were obtained from four independent experiments. Scale bar: 5 µm. The corresponding video is Movie S5.

**Figure 6:**
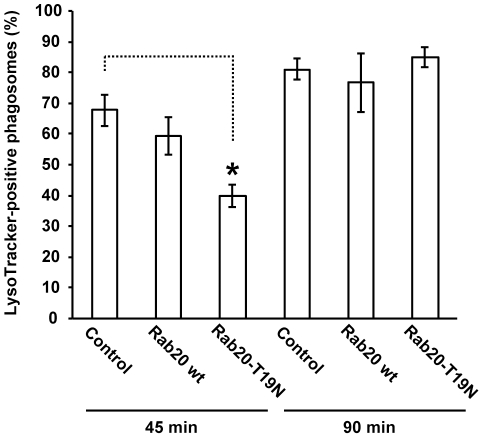
Expression of GDP-locked mutant Rab20-T19N abrogates phagosomal acidification. Quantification of LysoTracker colocalization with internalized phagosomes in cells expressing Rab20 wt or Rab20-T19N was conducted. The number of formed phagosomes and LysoTracker-positive phagosomes was counted under a microscope. The results are expressed as a percentage of LysoTracker-positive phagosomes. The data are means ± SEM of four independent experiments. Student's t-test was used for statistical analysis. *P<0.05.

Phagosomal localization of Rab20 in the intermediate/late stage of phagosome maturation strongly implies that Rab20 might be involved in phagolysosome formation. To explore the role of Rab20 in phagosome maturation, we first co-transfected RAW264 macrophages with CFP-Lamp1 and GFP-Rab20-T19N, and observed the delivery of Lamp1 to formed phagosomes in live cells expressing GDP-bound mutant of Rab20. Prior to the onset of phagocytosis, the distribution of GFP-Rab20-T19N in RAW264 cells is diffuse in the cytosol. Even after the addition of IgG-Es, no alternation in the localization of GFP-Rab20-T19N was observed. Moreover, the distribution of Lamp1-positive vesicles, namely late endosomes/lysosomes, was not affected by the expression of Rab20-T19N. After addition of IgG-Es to the cells, the uptake of IgG-Es normally occurred in RAW264 macrophages expressing GDP-locked mutant Rab20-T19N (Figure 5, Movie S5 and Figure S3). Importantly, we observed that the accumulation of Lamp1 in internalized phagosomes was significantly delayed in cells expressing GDP-locked mutant Rab20-T19N compared to non-expressing controls (Figure 5 and Movie S5). These observations indicate that the expression of GDP-bound mutant of Rab20 inhibits delivery of Lamp1 to phagosomes. Next, we investigated the effect of Rab20-T19N expression on the recruitment of Rab7 to internalized phagosomes. Time-lapse observation also showed that the expression of GDP-bound mutant Rab20-T19N inhibited Rab7 accumulation around the phagosomes (Figure S4). To quantitatively evaluate the effect of Rab20-T19N expression on phagosome maturation, we have employed LysoTracker Red, a marker for acidified compartments such as late endosomes and lysosomes. Cells expressing GFP-Rab20-T19N were allowed to ingest IgG-Es for 45 and 90 min in the presence of LysoTracker Red, then acidified phagosomes was quantified by scoring the number of phagosomes labeled with LysoTracker Red per internalized phagosomes under phase-contrast and fluorescence microscope. As shown in Figure 6, the expression of GDP-bound mutant Rab20-T19N delayed phagosomal acidification, whereas expression of Rab20 wt had no effect on phagosome maturation. These data suggest that Rab20 regulates the process of phagosome maturation during FcγR-mediated phagocytosis.

## Discussion

Our study provides the first evidence that the onset of Rab20 recruitment to phagosomes occurs slightly later than that of Rab5, although Rab20 and Rab5 are transiently colocalized on the internalized phagosomes. It has been shown that Rab5 is involved in the fusion of formed phagosomes with sorting endosomes and regulates phagosome maturation, and that expression of GDP-bound mutant of Rab5 has no effects on phagocytic efficiency [15]. We revealed that the expression of GDP- locked mutant of Rab20 did not inhibit FcγR-mediated phagosome formation. Therefore, it is unlikely that Rab20 is engaged in phagocytic cup formation and subsequent phagosome formation.

In this study, we found that Rab20 is recruited to the phagosomes decorated with Rab5 or Rab7. Moreover, our time-lapse imaging showed that Lamp1 acquisition by phagosomes is delayed in cells expressing GDP-locked mutant Rab20-T19N. Vieira *et al.* demonstrated that the number of Lamp1-positive phagosomes is reduced by expression of GDP-bound mutant of Rab5 [15]. Taking these facts into account, we conclude that Rab20 as well as Rab5 are involved in phagosome maturation, although the phagosomal localization of Rab20 is marginally different from that of Rab5. Like other Rab family members, the GDP-locked mutant of Rab20 functions as a dominant-negative mutant. Rab20-T19N potentially interacts with endogenous guanine nucleotide exchange factors (GEFs). So, it is assumed that the expression of Rab20-T19N could sequester the GEFs, inhibiting the activation of endogenous Rab20. To date, the downstream effector of Rab20 is unknown. So, precise mechanism for Rab20 function during phagosome maturation remains to be clarified. Importantly, Huynh *et al.* demonstrated that Lamp proteins are required for fusion of lysosomes and phagosomes [26]. In their study, Lamp-deficient phagosomes acquire Rab5, but fail to accumulate Rab7 and do not fuse with lysosomes. We observed that the recruitment of Rab7, as well as Lamp1, to nascent phagosomes is inhibited in Rab20-T19N expressing cells. Therefore, it is postulated that Rab20 might facilitate phagosome maturation via the recruitments of Lamp1 and Rab7. Moreover, our observation using LysoTracker Red showed that the expression of Rab20-T19N (GDP-locked mutant) significantly impacts on the acidification of phagosomes. Since Rab20 is localized with vacuolar H^+^-ATPase (V-ATPase) [22], this GTPase may be alternatively involved in the regulation of V-ATPase traffic during phagosome maturation.

At present, the functional molecules regulating Rab20 translocation to phagosomes remain to be elucidated. Rab20 is a close homologue of Rab5 [27] and colocalizes with Rab5-positive compartments (Figure 2). Interestingly, our microscopic observations revealed that Rab20 and Rab5 show similar but not identical spatiotemporal dynamics during FcγR-mediated phagocytosis. As described above, the association of Rab20 with phagosomes is preceded by that of Rab5 and persists even after Rab5 dissociation. These facts imply that the different GEFs and GTPase-activating proteins (GAPs) are involved in the regulation of Rab20 and Rab5 translocation. In further studies, the identification of GEFs, GAPs and downstream effectors of Rab20 would be important for understanding the physiological consequences of Rab20 in the sequential activation of Rab GTPases during phagosome maturation.

In conclusion, our study demonstrated that Rab20 is temporarily associated with phagosomes at a stage partially overlapping both the Rab5- and Rab7-functional stage, and is involved in the process of phagosome maturation during FcγR-mediated phagocytosis. The phagocytic pathway is coordinately regulated by several Rab GTPases, creating sequential Rab-effector recruitments that mediate the progressive maturation of the phagosomes.

## Materials and Methods

### Reagents

Bovine serum albumin (BSA) and Dulbecco's modified Eagle's medium (DMEM) were obtained from Sigma Chemical (St Louis, MO). Fetal bovine serum (FBS) was purchased from BioSolution International (Melbourne, Australia). LysoTracker Red DND-99 was obtained from Molecular Probes (Eugene, OR, USA). Other reagents were purchased from Wako Pure Chemicals (Osaka, Japan) or Nakalai tesque (Kyoto, Japan), unless otherwise indicated.

### Cell culture

Mouse macrophage RAW264 cells were cultured in DMEM supplemented with 10% heat-inactivated fetal bovine serum (FBS), 100 U/ml penicillin and 100 µg/ml streptomycin, as described in the manuals of cell line bank. Before the live-cell imaging, the culture medium was replaced with Ringer's buffer (RB).

### DNA constructs and transfection

The cDNA fragment comprising the entire coding region for human RAB20 was generated by PCR amplification of human cDNAs. The primers used were TCTCGAGCTATGAGGAAGCCCGACAGCAA and AGAATTCTCAGGCACAACACCCAGATC. The fragment was cloned into the XhoI and EcoRI restriction sites of the pEGFP-C1 and pECFP-C1 vector (Clontech, Palo Alto, CA). pEGFP-Rab20-T19N (GDP-bound mutant) was generated by means of the QuickChange II site-directed mutagenesis kit (Stratagene, La Jolla, CA). pEYFP-Rab5 and pEYFP-Rab7 were kindly provided by Dr. Joel A. Swanson (University of Michigan, Ann Arbor, MI). pECFP-Lamp1 was described previously [28]. pEGFP-Rab5 was generated by the replacement of YFP with GFP. pECFP-Rab7 was generated by the exchange of YFP. All constructs were verified by sequencing prior to use. Transfection of plasmids into RAW264 macrophages with Neon Transfection System (Invitrogen, Carlsbad, CA) was performed according to the manufacturer's instruction. The transfected cells were seeded onto 25-mm coverslips and maintained in the growth medium. Experiments were performed 24–48 h after transfection.

### RT-PCR

Total RNAs were isolated using a standard acid guanidinium thiocyanate phenol/chloroform extraction protocol. Total RNAs (5 µg aliquots) were converted to cDNAs with Superscript reverse transcriptase (Invitrogen, Gaithersburg, MD) and oligo(dT) nucleotides. PCR analysis was performed using specific primers. The primers used were follows: for Rab20, primers targeted bases 1–21 and 681–702 of GenBank Accession No. NM011227; and for GAPDH, primer targeted base 178–199 and 537–557 of GenBank Accession No. BC096440. Numbers of amplifying cycles were 34 for detection of Rab20, and 21 for detection of GAPDH. Reaction products were separated electrophoretically on a 1% agarose gel and visualized by staining with ethidium bromide.

### Live-cell imaging and image analysis

RAW264 cells were cultured on 25-mm circular coverslips and assembled in a RB-filled chamber on the thermo-controlled stage (Tokai Hit INU-ONI, Shizuoka, Japan) of an inverted epifluorescence microscope (Nikon TE300). Phase-contrast and fluorescence images of live cells were sequentially taken through a digital cooled CCD camera (Retiga Exi, QImaging, Surrey, BC, Canada) using shutters and filter wheels controlled by the MetaMorph imaging system (Molecular Devices, Downingtown, PA). In the cells co-expressing GFP- and CFP-fusion proteins, GFP and CFP images were acquired using a YFP filter set of excitation 505 nm and emission 540 nm and a CFP set of excitation 435 nm and emission 490 nm, respectively, to separate one signal from another. We confirmed that neither fluorescence signal was detected through the other filter set. Time-lapse images of phase-contrast and fluorescence microscopy were taken at 10 s intervals and assembled into QuickTime movies by the MetaMorph imaging system, as previously described [29]. At least 4 examples were observed in each experiment. For quantitative image analysis of fusion protein levels during phagocytosis, we defined time 0 as the moment when IgG-E binds to the cell surface. Then, we measured the fluorescent intensity in the phagocytic membranes, which were manually selected every 2 min throughout the time course after particle attachment. After background (cytoplasmic signal) subtraction, the time course of fluorescent intensity of each protein was plotted. The data are means ± SEM of four independent experiments.

### Phagocytosis and phagosome maturation

Seep erythrocytes were opsonized with rabbit anti-sheep erythrocyte IgG (1∶250, Organo Teknika-Cappel) and resuspended in PBS as described previously [5]. For the quantitative assay of phagocytosis, IgG-opsonized erythrocytes (IgG-Es) were added to adherent RAW264 macrophages. After 20 min of incubation with IgG-Es at 37°C, the cells on the coverslips were dipped into distilled water for 2 min to disrupt the extracellularlly exposed IgG-Es, and were fixed with 4% paraformaldehyde and 0.1% glutaraldehyde. The number of internalized IgG-Es was counted in 50 cells randomly chosen under a phase-contrast and fluorescent microscope. The phagocytic index, i.e. the mean number of IgG-Es taken up per cell, was calculated. The data are means ± SEM of four independent experiments. Student's t-test was used for statistical analysis. To estimate phagosome maturation, RAW264 macrophages were incubated for 45 and 90 min with 200 nM LysoTracker Red DND-99 in the presence of IgG-Es at 37°C. Following the incubation, the cells were fixed with 4% paraformaldehyde on coverslips. Phagosomes uniformly labeled with LysoTracker Red were scored positive. The number of internalized IgG-Es and LysoTracker-positive phagosomes (more than 50 phagosomes) was counted. The phagosome maturation index, i.e., the percentage of LysoTracker-positive phagosomes, was calculated. The data are means ± SEM of four independent experiments. Student's t-test was used for statistical analysis.

## Supporting Information

Movie S1 Time-lapse movie showing the localization of GFP-Rab20 during phagocytosis of IgG-Es. Note the association of Rab20 with newly formed phagosomes. The images were collected at 10-s intervals. This is a representative of four cells, and similar results were obtained from four independent experiments. This movie corresponds to Figure 1.
(MOV)

Movie S2 Time-lapse movie showing the dynamics of Rab20 and Rab5 during FcγR-mediated phagocytosis. RAW264 cells co-transfected with CFP-Rab20 (red) and GFP-Rab5 (green) were put into contact with IgG-Es. The GFP and CFP images were acquired every 10 s. This movie corresponds to Figure 2.
(MOV)

Movie S3 Time-lapse movie showing the dynamics of Rab20 and Rab7 during phagocytosis of IgG-Es. The time-lapse images of RAW264 macrophages co-expressing CFP-Rab20 (red) and YFP-Rab7 (green) were taken every 10 s. This movie corresponds to Figure 3, which is a representative of four experiments.
(MOV)

Movie S4 Time-lapse movie showing the dynamics of Rab20 and Lamp1 during FcγR-mediated phagocytosis. RAW264 macrophages co-expressing GFP-Rab20 (green) and CFP-Lamp1 (red) were incubated with IgG-Es. The GFP and CFP images were acquired every 10 s. The movie corresponds to Figure 4.
(MOV)

Movie S5 Time-lapse movie showing Lamp1 localization in a Rab20-T19N expressing cell (upper cell) and a non-expressing cell (lower cell). RAW264 cells co- transfected GFP-Rab20-T19N (left) and CFP-Lamp1 (right) were put into contact with IgG-Es. The GFP and CFP images were acquired every 10 s. The movie corresponds to Figure 5.
(MOV)

Figure S1 Expression of Rab20 mRNA in RAW264 cells and bone-marrow-derived macrophages. RT-PCR assay of Rab20 mRNA in RAW264 cells, bone-marrow-derived macrophages, NIH3T3 cells and C2C12 myoblasts was performed. Expression of GAPDH mRNA was used as an internal control. It is noteworthy that Rab20 is predominantly expressed in RAW264 cells and bone-marrow-derived macrophages. Similar results were obtained from four independent experiments.
(TIF)

Figure S2 Changes in Rab5, Rab20, Rab7 and Lamp1 levels on individual phagosomes. The amounts of Rab5, Rab20, Rab7 and Lamp1 on nascent phagosomes were quantified by image analysis of fluorescent intensities of each protein. The data are means ± SEM of four independent experiments. The y-axis units are arbitrary.
(TIF)

Figure S3 Quantification of phagocytosis in RAW264 cells expressing wild-type Rab20 or Rab20-T19N. Phagocytosis of IgG-Es by RAW264 macrophages expressing wild-type GFP-Rab20 or Rab20-T19N were compared with control untransfected cells. The results are expressed as phagocytic index. The data are means ± SEM of four independent experiments. Student's t-test was used for statistical analysis. There was no statistically significant difference in the phagocytic index between cells expressing GFP-Rab20 or Rab20-T19N and the control cells.
(TIF)

Figure S4 Time-lapse images showing different dynamics of Rab7 between a Rab20-T19N-expressing cell and a non-expressing cell during phagocytosis of IgG-Es. RAW264 cells co-expressing GFP-Rab20-T19N and CFP-Rab7 were exposed to IgG-Es and observed by phase-contrast and fluorescence microscopy. The recruitment of Rab7 to formed phagosomes was inhibited in cells expressing Rab20-T19N (arrows) as compared to non-expressing controls (arrowheads). Representative images from three independent experiments are shown. Scale bar: 5 µm.
(TIF)
