# Supplementary figures and images for: Rab20 Regulates Phagosome Maturation in RAW264 Macrophages during Fc Gamma Receptor-Mediated Phagocytosis
Source: PLoS One. 2012 Apr 24;7(4):e35663. doi: 10.1371/journal.pone.0035663 (PMC3335809; doi:10.1371/journal.pone.0035663)

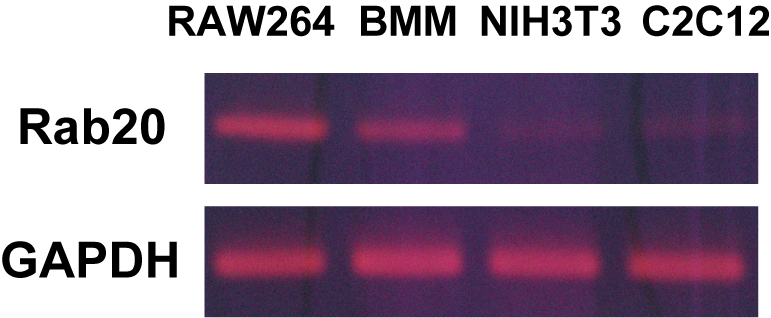

Supplement: Figure S1 — Expression of Rab20 mRNA in RAW264 cells and bone-marrow-derived macrophages. RT-PCR assay of Rab20 mRNA in RAW264 cells, bone-marrow-derived macrophages, NIH3T3 cells and C2C12 myoblasts was performed. Expression of GAPDH mRNA was used as an internal control. It is noteworthy that Rab20 is predominantly expressed in RAW264 cells and bone-marrow-derived macrophages. Similar results were obtained from four independent experiments. (TIF) [file pone.0035663.s006.tif]

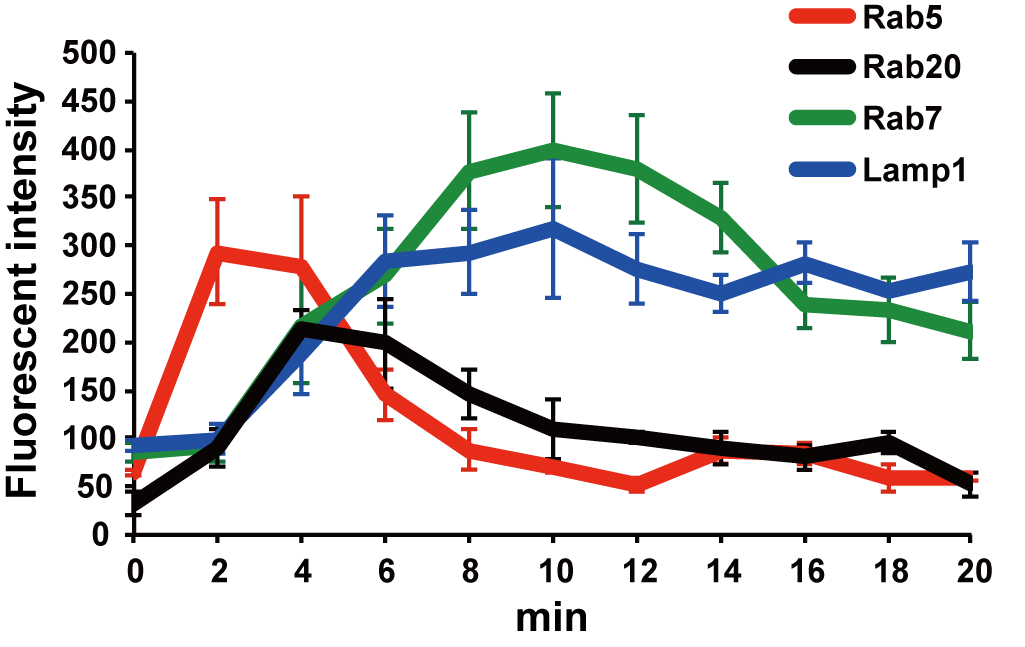

Supplement: Figure S2 — Changes in Rab5, Rab20, Rab7 and Lamp1 levels on individual phagosomes. The amounts of Rab5, Rab20, Rab7 and Lamp1 on nascent phagosomes were quantified by image analysis of fluorescent intensities of each protein. The data are means ± SEM of four independent experiments. The y-axis units are arbitrary. (TIF) [file pone.0035663.s007.tif]

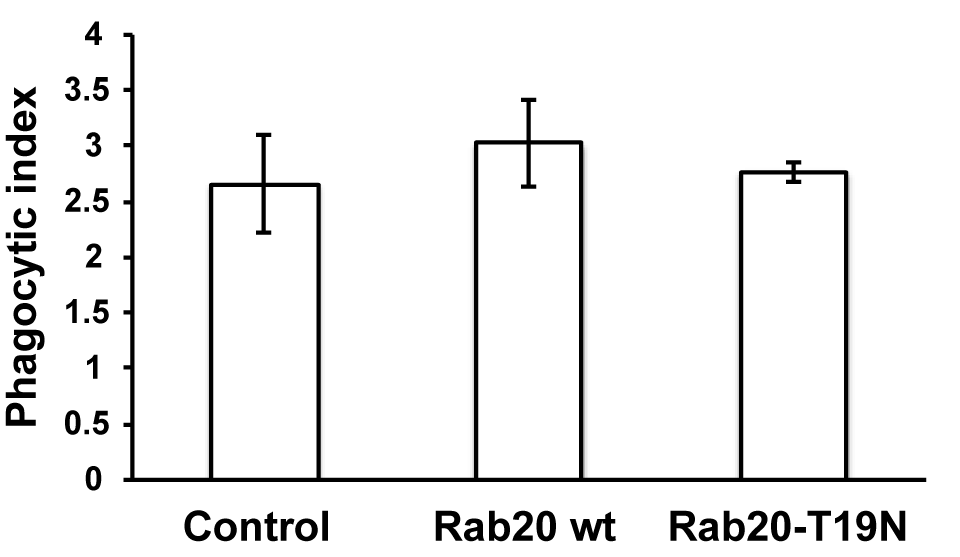

Supplement: Figure S3 — Quantification of phagocytosis in RAW264 cells expressing wild-type Rab20 or Rab20-T19N. Phagocytosis of IgG-Es by RAW264 macrophages expressing wild-type GFP-Rab20 or Rab20-T19N were compared with control untransfected cells. The results are expressed as phagocytic index. The data are means ± SEM of four independent experiments. Student's t-test was used for statistical analysis. There was no statistically significant difference in the phagocytic index between cells expressing GFP-Rab20 or Rab20-T19N and the control cells. (TIF) [file pone.0035663.s008.tif]

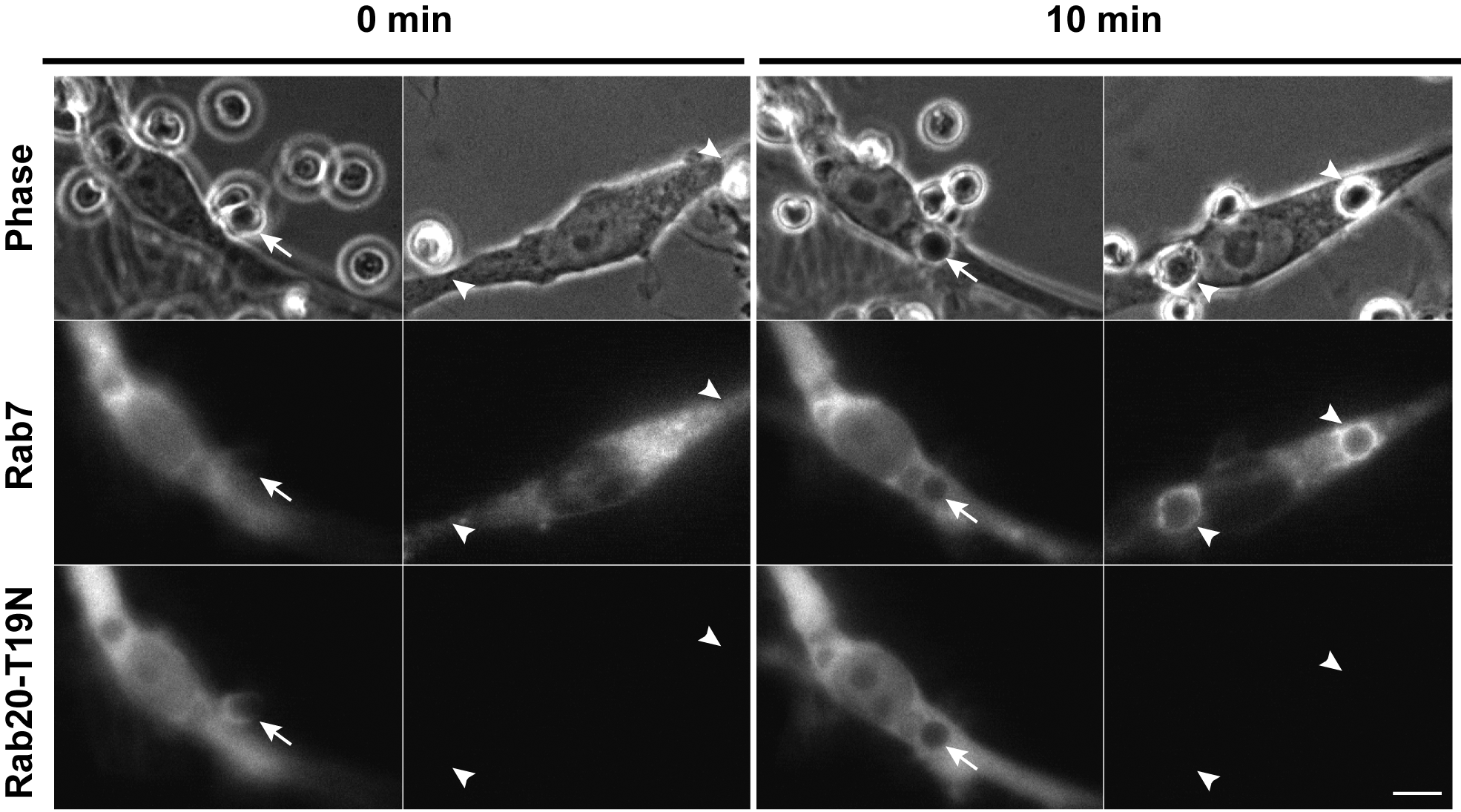

Supplement: Figure S4 — Time-lapse images showing different dynamics of Rab7 between a Rab20-T19N-expressing cell and a non-expressing cell during phagocytosis of IgG-Es. RAW264 cells co-expressing GFP-Rab20-T19N and CFP-Rab7 were exposed to IgG-Es and observed by phase-contrast and fluorescence microscopy. The recruitment of Rab7 to formed phagosomes was inhibited in cells expressing Rab20-T19N (arrows) as compared to non-expressing controls (arrowheads). Representative images from three independent experiments are shown. Scale bar: 5 µm. (TIF) [file pone.0035663.s009.tif]
